# Supplementary material for: The Apoptosis Inhibitor Protein Survivin Is a Critical Cytoprotective Resistor against Silica-Based Nanotoxicity
Source: Nanomaterials (Basel). 2023 Sep 12;13(18):2546. doi: 10.3390/nano13182546 (PMC10535920; doi:10.3390/nano13182546)
Supplement: Supplementary file 1 [file nanomaterials-13-02546-s001.zip › nanomaterials-2595985-supplementary.pdf]

Supplementary information

The apoptosis inhibitor protein Survivin is a critical cytoprotective resistor against silica-based nanotoxicity

Christina Breder-Bonk[1*], Dominic Docter[1], Matthias Barz[2,3], Sebastian Strieth[4], Shirley Knauer[5,] Désirée Gül[1*], and Roland H. Stauber[1]

Tables

Table S1. Characterization of amorphous silica nanoparticle. The size of SiNP of three different types was measured with TEM. Dynamic light scattering analysis (DLS) was performed in various aqueous solutions to determine the Zeta potential as well as size, size distribution and shape.

	**aSiNP**	**aSiNPL**	**aSiNP125**
R_{TEM}.[nm]	15.7 ± 1.9	15.6 ± 2.0	54.9 ± 17.2
NP conc. stock solution.[p/mL]	$5.72 \cdot 10^{16}$	$5.20 \cdot 10^{16}$	$7,43 \cdot 10^{14}$
SiO_2 conc. stock solution.[mg/mL]	575	667	573
**Milli-Q-water:**			
Zeta potential.[mV]	-51	-57	-57
R_h.[nm]	16.4	16.0	72.0
μ^2	0.12	0.06	0.03
**PBS:**			
Zeta potential.[mV]	-18	-20	-23
R_h.[nm]	18.4	17.1	80.8
μ^2	0.08	0.04	0.03
**RPMI 1640:**			
Zeta potential.[mV]	-20	-27	-23
R_h.[nm]	17.7	16.1	71.3
μ^2	0.07	0.02	0.05

Table S2. Characterization of the nanoparticle stability in different aqueous solutions over time. Two types of silica nanoparticles were characterized with DLS after incubation in Milli-Q-water, PBS or RPMI 1640 Medium.

	**aSiNP**			**aSiNPL**	
**Time:**	0h	2h	4h	0h	48h
**Milli-Q-water:**					
R_h.[nm]	16.4	16.1	16.3	16.0	15.7
μ^2	0.12	0.11	0.07	0.06	0.03
**PBS:**					
R_h.[nm]	18.4	18.0	17.8	17.1	16.3
μ^2	0.08	0.03	0.09	0.04	0.03
**RPMI 1640:**					
R_h.[nm]	17.7	17.4	17.6	16.1	17.7
μ^2	0.07	0.09	0.07	0.02	0.06

Figures

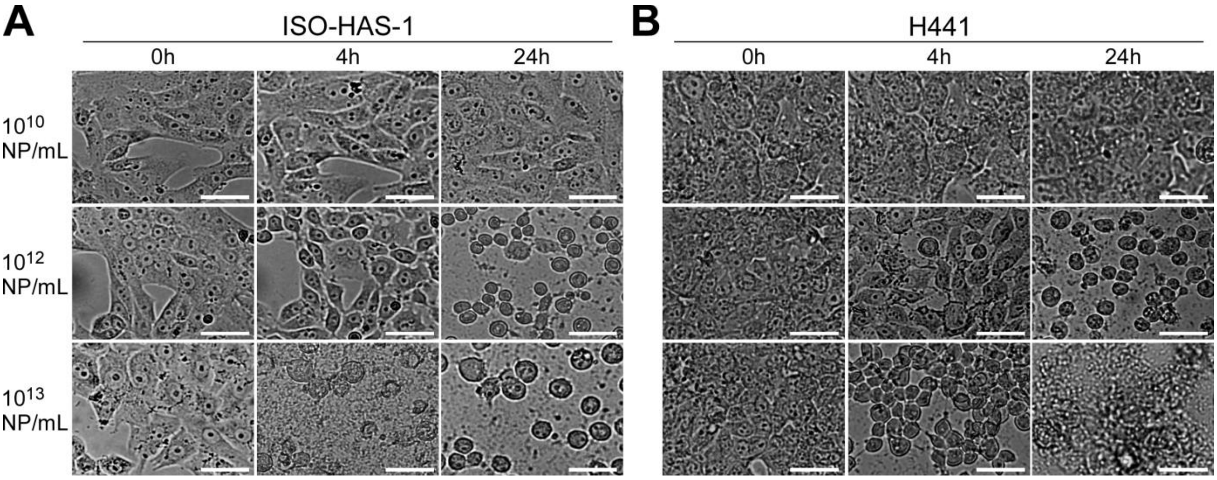

Supplementary figure S1. ISO-HAS-1 and H441 cells were treated with different particle concentrations in serum free medium for 4 h. For later time points treated cells were cultured for additional 20 h in serum containing medium. Morphological changes caused by SiNP (NexSil20) in ISO-HAS-1 (**A**) and H441 cell line (**B**). Scale bar indicates for 150µm.

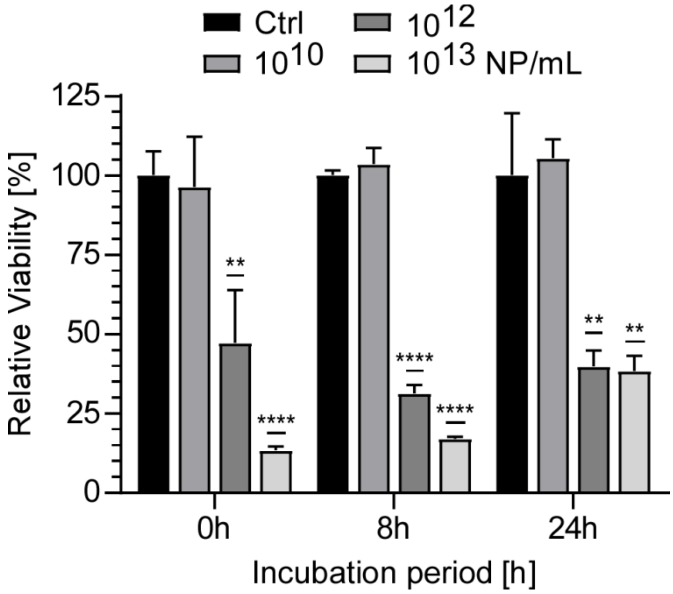

Supplementary figure S2. Impact of aSiNP L on ISO-HAS-1 cells. ISO-HAS-1 treated with different particle concentrations in serum free medium for 4h. For later time points treated cells were cultivated for additional 20 h in serum containing medium. Viability was measured via celltiter glow viability assay. Three independent experiments were performed and data normalized to control groups. Unpaired t-test was used to compare treated cells to untreated control groups. Column, mean; bars, ± S.D. **, $p < 0.001$, ****, $p < 0.0001$.

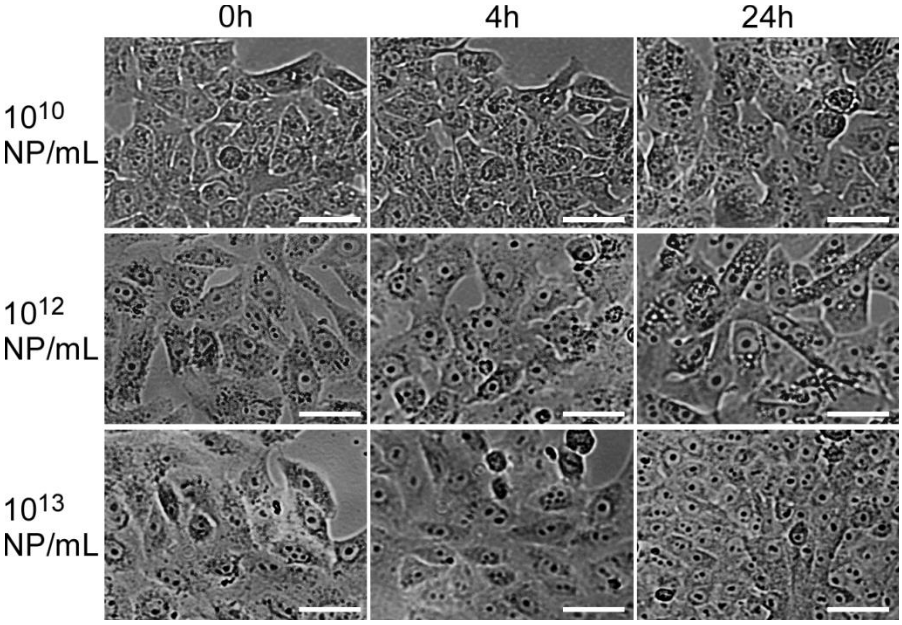

Supplementary figure S3. Effect of amorphous silica nanoparticle with a diameter of 125 nm (aSiNP125). H441 cells treated with increasing aSiNP125 concentrations, while cells were treated for 4h in serum deprived medium with additional incubation in full growth medium for additional 20 h. Morphological changes were captured after 0 h, 4 h and 24 h.

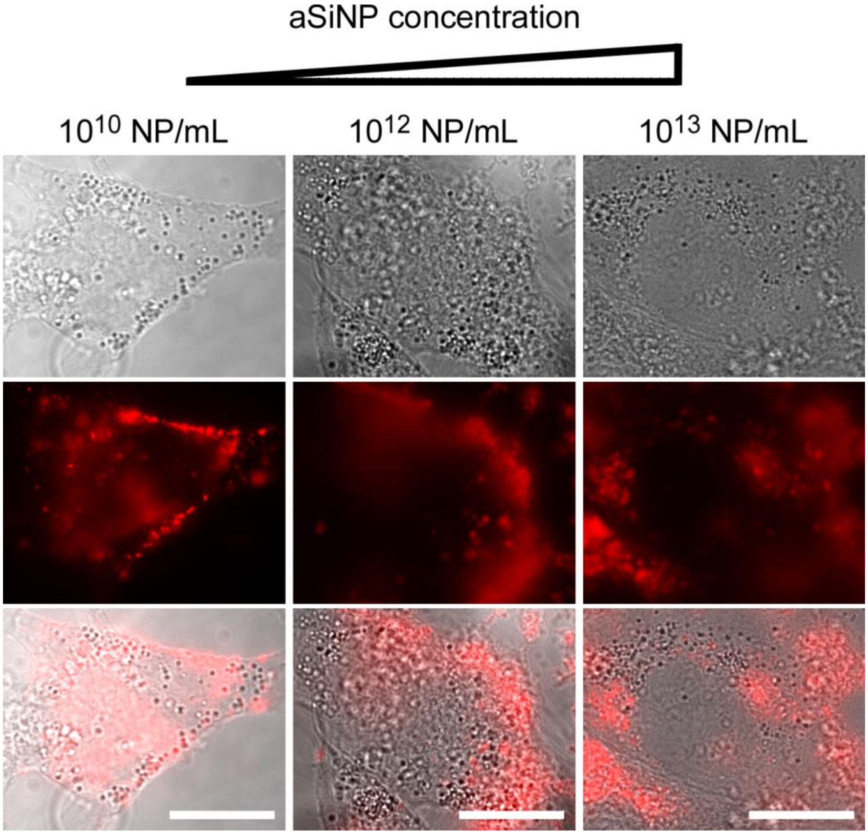

Supplementary figure S4. Interaction and internalization of aSiNPs with an increased diameter of 200nm by epithelial lung cells. H441 cells were incubated with increasing concentrations of fluorescent aSiNP of type Kisker red (200nm) for 2 h in serum deprived medium. Scale bars indicate for 50 µm.

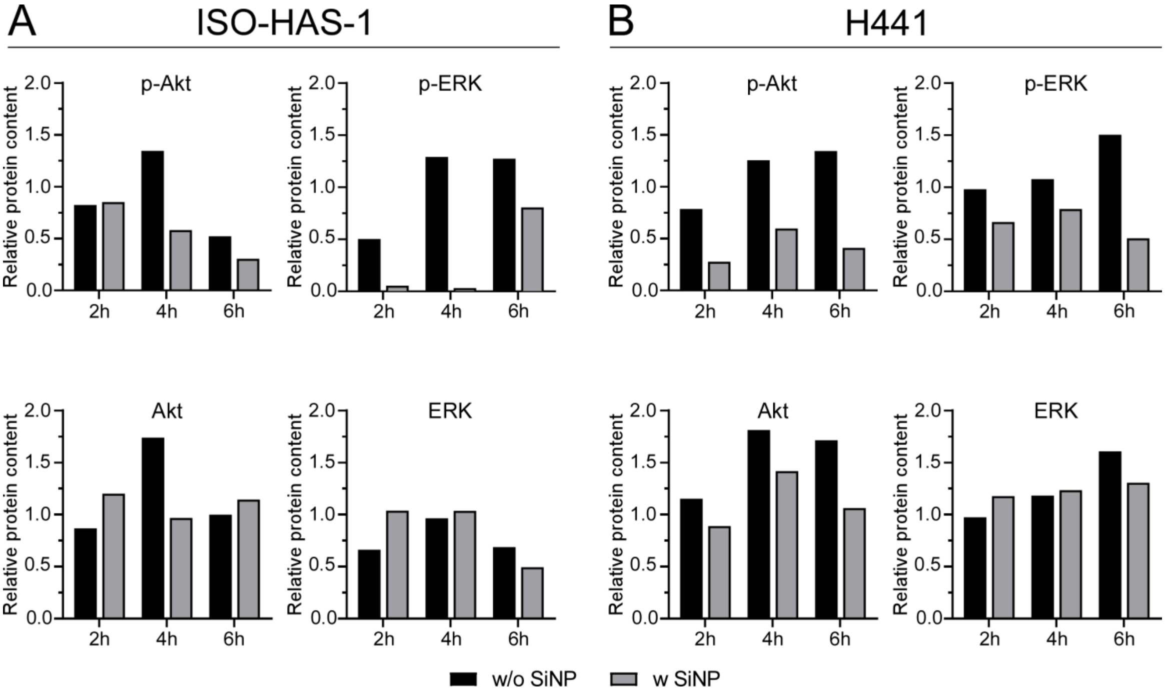

Supplementary figure S5. Quantification of protein bands detected via western blot analysis shown in Figure 5 by using Image J software. Data normalized to housekeeping gene actin. Akt and ERK signaling examined in ISO-HAS-1 cells (**A**) and H441 cells (**B**).

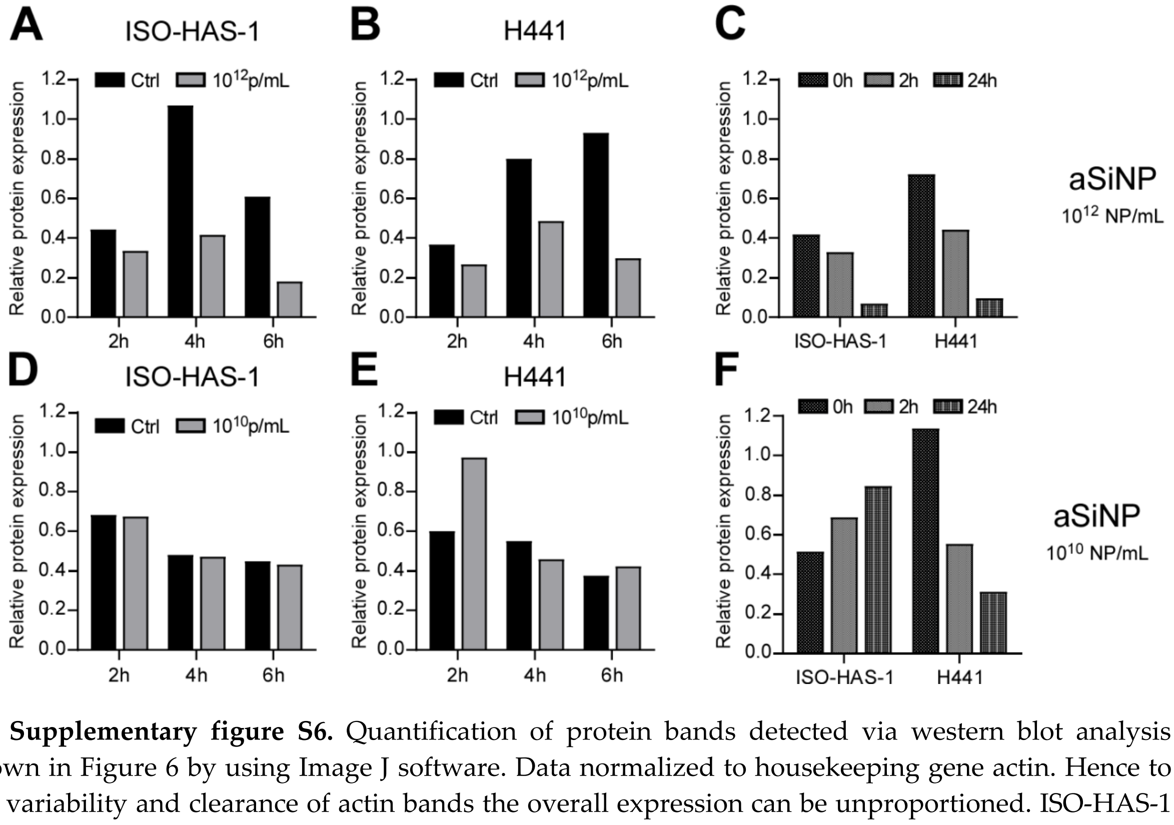

Supplementary figure S6. Quantification of protein bands detected via western blot analysis shown in Figure 6 by using Image J software. Data normalized to housekeeping gene actin. Hence to the variability and clearance of actin bands the overall expression can be unproportioned. ISO-HAS-1 (**A**) and H441 cells (**B**) exposure to 10^{12} NP/mL expressed survivin levels. Survivin protein expression after long term exposure to 10^{12} NP/mL shown for both cell lines in (**C**). Survivin protein expression quantified after exposure to 10^{10} NP/mL in ISO-HAS-1 (**D**) or in H441 (**E**) as well as long time exposure of 10^{10} NP/mL to both cell lines (**F**).

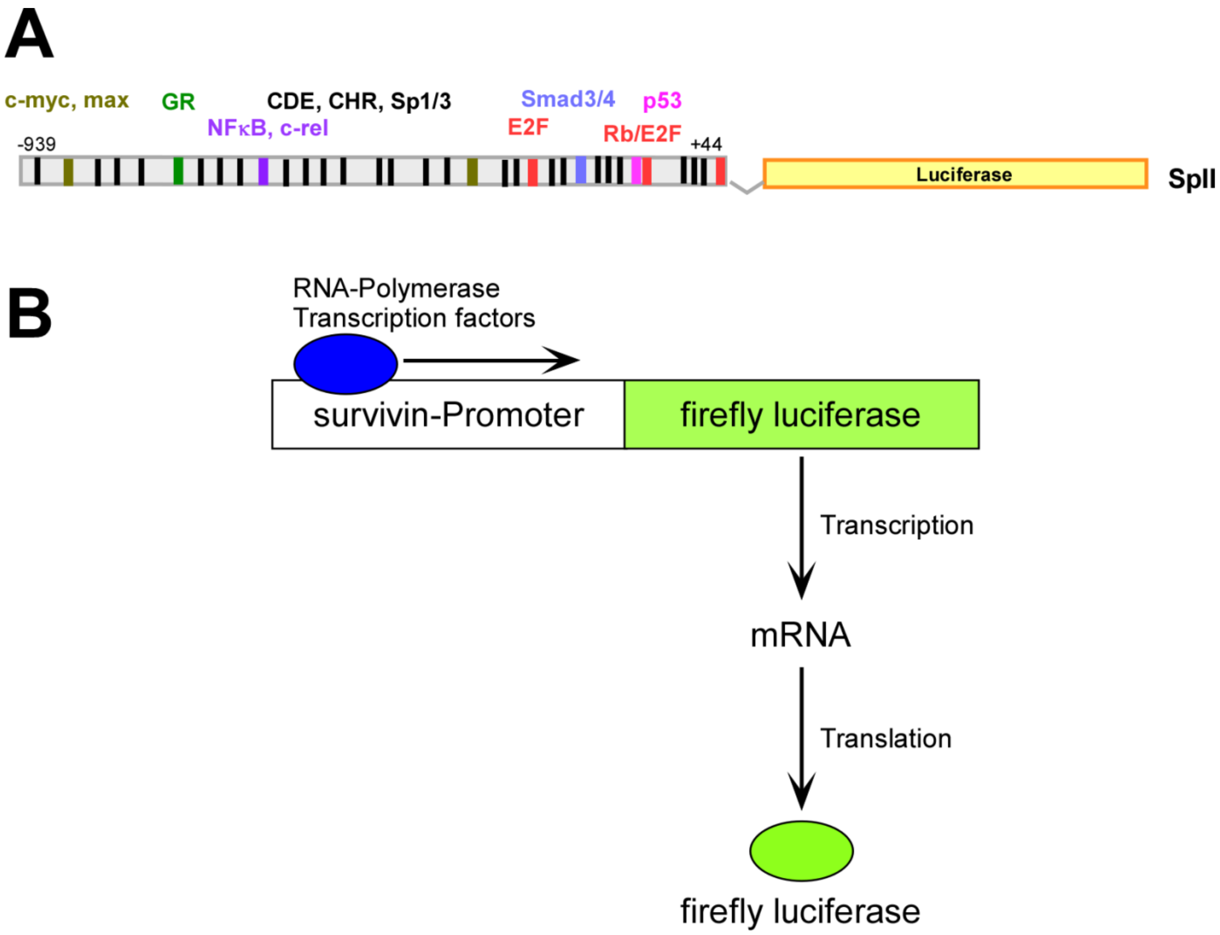

Supplementary figure S7 Luciferase Reporter Assay. Gene construct used in the Luciferase Reporter Assay (A). Functionality of the Luciferase Reporter Assay (B).
